# Nanoparticles for Catalysis

**DOI:** 10.3390/nano6070123

**Published:** 2016-06-23

**Authors:** Sergio Navalón, H. García

**Affiliations:** 1 Deparment of Chemistry, Universidad Politécnica de Valencia, C/Camino de Vera, s/n, 46022 Valencia, Spain; sernaol@doctor.upv.es; 2 Instituto Universitario de Tecnología Química CSIC-UPV, Universitat Politécnica de Valencia, Av. De los Naranjos s/n, 46022 Valencia, Spain; 3 Center of Excellence for Advanced Materials Research, King Abdulaziz University, 21589 Jeddah, Saudi Arabia

Nanoscience emerged in the last decades of the 20th century with the general aim to determine those properties that appear when small particles of nanometric dimensions are prepared and stabilized. One of the clearest examples of specific properties of nanoparticles (NPs) is the ability to catalyze reactions by interacting with substrates and reagents. These unique properties of NPs as catalysts derive from the large percentage of coordinatively unsaturated atoms located at the surface, edges and corners of the NPs compared to the total number of atoms. Particularly those atoms located at steps, corners and edges of NPs exhibit the highest catalytic activity due to their low coordination number and their high tendency to increase this number by coordinating with substrates and other species in the surroundings.

While NPs can be prepared by different procedures, their main drawback is their tendency to undergo agglomeration as they increase in size, thereby reducing the energy associated with large surface area. One general methodology to circumvent this problem is by adsorbing these NPs on large surface area of insoluble solids that by means of strong interactions are able to adsorb NPs on their surface, stabilizing them against sintering and growth. In addition to this role, supports can also play an additional role in the catalysis providing acidity/basicity or by tuning the electronic density of the NPs.

The present Special Issue of Nanomaterials falls with the domain of NPs applied to catalysis. In their review, Palomo and Filice illustrate the synthesis of metal NPs by reduction of the corresponding metal precursors using biomolecules, plant extracts and even microorganisms [1]. The general advantage of these methods to form metal NPs is that no chemical reducing agents are employed and, in this sense, the synthesis based on the use of biomolecules can be environmentally more benign and sustainable, resulting in the generation of fewer chemical residues. In addition, biomolecules can also act as ligands of the metal NPs and, in this way, they not only reduce the metal ions to the metallic state, but also contribute to the formation of stable suspensions in water or other green solvents.

As discussed above, the solid support adsorbing NPs should primarily increase the stability of otherwise highly reactive NPs. In their study, Doris and co-workers have used carbon nanotubes (CNTs) as scaffolds to deposit a particular organic polymer on which gold NPs can be deposited [2]. Specifically, CNTs were covered by the polymer resulting from light-promoted nitrilotriacetic-diyne polymerization followed by subsequent Au deposition. The resulting construct has a defined 1D morphology imparted by the CNT scaffold and was used to catalyze the aerobic oxidation of hydroxylamines to nitrones. Besides thermal oxidations, NPs exhibit interesting properties as photocatalysts. This aspect has also been covered in this Special Issue with two contributions showing the activity of small TiO_2_ for the removal of pollutants [3] and for the selective oxidation of benzylalcohol to benzaldehyde [4].

In addition to oxidations, NPs, particularly metal NPs, have also general catalytic activity in reductions. In one of the articles of this Special Issue, van der Voort and co-workers have encapsulated Pt NPs inside the cages of a metal-organic framework (MIL-101-Cr) by atomic layer deposition [5]. Due to their internal porosity and crystalline structure, metal-organic frameworks offer confined spaces with regular dimensions in which NPs can be stabilized by geometrical constrains. Besides molecular hydrogen, other reducing agents such as hydrazine [6], NaBH_4_ [7,8,9] and aminoborane [7] can also be activated by metal NPs to promote the reduction of different compounds. Nitroaromatics are among the preferred model substrates when the purpose is to determine the activity of catalysts, due to the possibility to follow the course of the reaction by absorption spectroscopic [7,8,9]. As in the case of oxidations, reductions can also be promoted by light. In one of these examples, Jain and co-workers have used a phosphorous-doped carbon nitride semiconductor that has been modified by Ni NPs and this system is able to promote light-assisted nitroarene to aniline reductions by using hydrazine as reducing agent [6].

Supported metal NPs are also suitable for use in the promotion of electrocatalytic reactions. Due to their interest in fuel cells, Qi and co-workers have prepared ultra-small size Pt NPs stabilized by polyvinylpyrrolidone that have been used as electrodes in methanol oxidation to CO_2_ [10]. This reaction is of great importance for low temperature fuel cells. In a related work, Li and co-workers have shown that N-doped TiO_2_ nanotubes as additives also increase the activity of graphene supported Pt composites in electrocatalytic methanol oxidation [11].

Continuing in the domain of renewable energies, NPs also offer considerable advantages with respect to other materials for the development of more efficient and cyclable electrodes for batteries. One type of battery that is very promising in the future due to high energy density and availability is the Li-O_2_ battery. One of the main limitations of this type of batteries is the development of an efficient catalyst for the electrochemical reactions involving gas-phase O_2_ and solid lithium oxides. In this context, Chen and co-workers have shown that MnO_2_ NPs supported on carbon composites exhibit a high catalytic activity for oxygen reduction reaction [12], one of the key reactions involved. Similarly, MnO_2_-doped with Ag dispersed in porous carbonaceous matrices are suitable gas diffusion electrodes for this type of batteries [13].

Overall, the present Special Issue shows the breath of applications and the potential of NPs in various fields going from thermal catalysis of liquid and gas phase reactions to photocatalysis using visible or solar light and electrocatalysis. In all these fields, the activities of nanomaterials have been found to exceed those of other types of particles. The target in this area is to further reduce the particle size, while gaining control of the morphology and facet orientation of the NPs, as well as their stabilization, without diminishing their activity. Another general challenge is to replace noble and critical metals by other abundant base transition metals. These targets will surely be accomplished in the near future.
